# 

**Published:** 1868-05

**Authors:** 


					﻿VOL. XXII.	No. 5.
THE
Dental lAegifilcn
z
> **
EDITED BY
J. TAFT & G. WATT.
MAY, 1868.
MONTHLY:
THREE DOLLARS PER ANNUM, IN ADVANCE.
CINCINNATI;
WRIGHTSON & CO., Printers, 167 WALNUT STREET.
J. A W2[. TAFT, Dentists, 238 West Fourth St.
				

